# An autophagic gene‐based signature to predict the survival of patients with low‐grade gliomas

**DOI:** 10.1002/cam4.3748

**Published:** 2021-02-16

**Authors:** Jian Chen, Yuntian Li, Xinghua Han, Yueyin Pan, Xiaojun Qian

**Affiliations:** ^1^ Oncology department The First Affiliated Hospital of USTC Division of Life Sciences and Medicine University of Science and Technology of China Hefei P.R. China

**Keywords:** autophagy, low‐grade gliomas, nomogram, prognosis

## Abstract

**Background:**

Since autophagy remains an important topic of investigation, the RNA‐sequence profiles of autophagy‐related genes (ARGs) can provide insights into predicting low‐grade gliomas (LGG) prognosis.

**Methods:**

The RNA‐seq profiles of autophagic genes and prognosis data of LGG were integrated from the Cancer Genome Atlas (TCGA) and Chinese Glioma Genome Atlas (CGGA). Univariate Cox analysis and the least absolute shrinkage and selection operator (LASSO) regression model were carried out to identify the differentially expressed prognostic autophagy‐related genes. Then, the autophagic‐gene signature was formed and verified in TCGA test set and external CGGA cohorts. Time‐dependent receiver operating characteristic (ROC) was examined to test the accuracy of this signature feature. A nomogram was conducted to meet the needs of clinicians. Sankey diagrams were performed to visualize the relationship between the multigene signatures and clinic‐pathological features.

**Results:**

Twenty‐four ARGs were finally identified most relevant to LGG prognosis. According to the specific prediction index formula, the patients were classified into low‐risk or high‐risk groups. Prognostic accuracy was proved by time‐dependent ROC analysis, with AUC 0.9, 0.93, and 0.876 at the survival time of 2‐, 3‐, and 5‐year, respectively, which was superior to the AUC of the isocitrate dehydrogenase (IDH) mutation. The result was confirmed while validated in the TCGA test set and external validation CGGA cohort. A nomogram was constructed to meet individual needs. With a visualization approach, Sankey diagrams show the relationship of the histological type, IDH status, and predict index. In TCGA and CGGA cohorts, both low‐risk groups displayed better survival rate in LGG while histological type and IDH status did not show consistency results.

**Conclusions:**

24‐ARGs may play crucial roles in the progression of LGG, and LGG patients were effectively divided into low‐risk and high‐risk groups according to prognostic prediction. Overall, our study will provide novel strategies for clinical applications.

## Graphical abstract

24‐ARGs may play crucial roles in the progression of LGG, and the signature based on 24‐ARGs can effectively classify LGG patients into low‐risk and high‐risk groups in terms of prognostic prediction. Overall, our study will provide novel strategies for clinical applications.

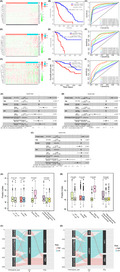

## 1 INTRODUCTION

Gliomas are the most frequently diagnosed brain tumors and account for over 30% of all primary brain and central nervous system (CNS) tumors in adults.^1^ Low‐grade gliomas (LGG), known as grade II glioma according to World Health Organization (WHO) classification, include three histological subtypes: astrocytoma, oligodendroglial, and oligoastrocytoma.^2^ Although LGGs are well‐differentiated, slowly growing and less aggressive, nearly half of the patients died after surgery due to recurrence or metastasis.^3^ In recent years, the prognosis of LGG patients has been improved significantly because of the developments of multidisciplinary treatments.^2^ However, great heterogeneity in prognosis has also been observed.^4^ It has been proposed that the survival of patients with LGG varies greatly, likely stems from the different genetic background. Therefore, it is very essential to identify molecular biomarkers to predict the prognosis and perform appropriate and individualized therapies.

IDH mutation is a recognized prognostic biomarker for LGG patients. Patients with IDH1 or IDH2 mutation tend to have superior survival than others.^5^ However, approximately 80%–90% of LGG patients harbor IDH1 and less than 1% harbor IDH2 mutations, which means that only a fraction of patients with poor or good prognosis can be identified based on IDH mutation status. MGMT promoter methylation level in initial tumors may be used to anticipate future aggressive clonal outgrowths of hypermutated and malignantly transformed tumor cells, while not exactly to predict the prognostic of LGG.^6^ NF‐kB‐p65 protein was identified as an independent predictor of both overall survival and malignant progression‐free survival in grade II LGG.^7^ Above all, a comprehensive and precise tool is expected to assist clinical procedure. The multigene signatures prediction classifier is an important method, which is currently used in the prediction of recurrence of multiple tumors, including colorectal cancer,^8^, ^9^, ^10^, ^11^ bladder cancer,^12^ laryngeal cancer,^13^ Lower Grade Glioma,^14^ etc.

Autophagy is an important mechanism in the processes of transporting damaged, denatured or aging proteins, digestion, and degradation of organelles.^15^ These processes are mediated by autophagy‐related genes (ARGs). Previous studies have identified more than 200 ARGs, directly or indirectly participating in the process of autophagy.^16^ Recently, it has also been demonstrated that autophagy is strongly related to tumor occurrence, inflammatory, therapeutic resistance, and cell death. Autophagy could inhibit migration and invasion of glioblastoma cells.^17^ Autophagy may be a paradigm of duality in hepatocarcinogenesis.^18^ Downregulated p62, which is a selective substrate for autophagy, related to the development of CRC.^19^ Moreover, several studies have demonstrated that some ARGs also related to the survival of patients with tumor and ARG‐based signatures could accurately predict the prognosis of patients.^16^, ^20^

However, the relationship between autophagy and low‐grade glioma has been rarely reported. More recently, it has been found that autophagy proteins were reduced and correlated with progression‐free survival in LGG.^21^, ^22^ Therefore, with the hypothesis that the malignancy‐risk gene signatures has prognostic and predictive value for LGG, the RNA‐seq profiles of ARGs and prognosis data of LGG were integrated from the Cancer Genome Atlas (TCGA) and the Chinese Glioma Genome Atlas (CGGA). Then, a predict risk formula was developed to predict the clinical outcome of patients with LGG. A total of 728 samples were used for statistical analysis. Clinical parameters included age, gender, IDH mutation status, histopathological types, and overall survival (Table 1). Utilizing the sample‐splitting method and Cox regression analysis, the prognostic predicts associated with 24‐ARGs can be determined from the TCGA training cohort and verified in the TCGA test cohort and CGGA cohort. Finally, according to the recurrence‐free survival rate, a nomogram was constructed as a quantitative prediction tool to assess clinical prognosis and assist clinical procedures.

**TABLE 1:** Clinicopathological features of LGG (low‐grade glioma) cases in TCGA and CGGA cohorts

Variables	TCGA cohort N=504			CGGA cohort *N* = 224
	Training set *N* = 352	Test set *N* = 152	*p*‐value	
Age (mean, range)	41 (17–87)	39 (14–75)	0.162	
Gender				
Male	199	81	0.565	134
Female	153	71		90
IDH				
Mutation	277	128	0.263	175
Wild	95	24		49
Histopathological				
Astrocytoma	128	63	0.525	66
Oligodendroglioma	131	54		49
Oligoastrocytoma	93	35		109
Overall survival status				
Alive	261	118	0.472	164
Death	91	34		60

## 2 MATERIALS AND METHODS

### 2.1 Data acquisition

The ARGs were obtained from the Human Autophagy Database (HADb, http://autophagy.lu/clustering/index.html).

The RNA‐sequencing (RNA‐seq) data of LGG cohorts as well as clinicopathological information were obtained from the TCGA (https://tcga‐data.nci.nih.gov/) and CGGA (http://www.cgga.org.cn) databases. The TCGA data set was randomly split into the training and internal test sets, and the CGGA data set severed as the external test set.

### 2.2 Construction of the ARGs prognostic model

Univariate Cox proportional hazards regression model was used in the training set to identify ARGs that were significantly correlated with overall survival for LGG patients and a total of 276 prognostic ARGs were identified. Then, the prognostic model based on these ARGs was constructed through LASSO regression, using the “glmnet” package in R 3.5.3.^23^ The penalty coefficient, namely lambda, was optimized through 10‐fold cross‐validation in the training set.

### 2.3 Evaluation of the prognostic model

Linear predict indexes (PIs) were calculated based on the model for all samples. The “survminer” package in R 3.5.3 was used to determine the cutoff value of the PI, which was used to classified patients into the high‐risk group and low‐risk group in the training, internal, and external test sets. The Log‐rank test was used to compare the overall survival between the patients in the high‐risk group and those in the low‐risk group. The univariate and multivariate Cox proportional hazard regression analysis, and time‐dependent receiver operating characteristic (ROC) curve were also used to access the predictive ability of the model. Finally, the subgroup analyses were performed to evaluate the robustness of the model. In addition, we developed a prognostic nomogram based the PI and clinicopathological characteristics using the “rms” package in R 3.5.3, and validated it through calibration curves.

### 2.4 Functional enrichment analysis

Exploration of the prognostic model to explore the potential mechanisms of the model, we identified the co‐expressed genes of ARGs in the model by the Pearson’s correlation test. Then, functional enrichment analyses including GO and KEGG annotations were performed based on the co‐expressed genes. Additionally, we also analyzed the correlations between the model and clinicopathological characteristics.

### 2.5 The correlation between predict index and clinic‐pathological parameters

The relationship between predict index and clinic‐pathological parameters were analyzed via box plots and Sankey diagrams by independent samples nonparametric tests in the TCGA cohort and CGGA cohort. Sankey diagrams were plotted using “ggforce” package in R.^24^ Kaplan–Meier survival curve analysis were performed to investigate the potential of predict index as prognostic factors in clinic‐pathological parameters based on TCGA and CGGA cohorts.

### 2.6 Statistical analysis

All statistical analysis involved were performed using R (version 3.5.1, www.r‐project.org). All statistical tests was two‐sided, and *p* < 0.05 was considered statistically significant.

## 3 RESULTS

### 3.1 Preparation of data sets

About 504 cases of LGG (low‐grade glioma) from the TCGA cohort were randomized into training and test sets by 0.7:0.3. Mortality differential in the two sets was the same. Two external validation series mRNAseq_693 and mRNAseq_325 involving 224 cases of LGG (low‐grade glioma) were from the CGGA cohort. Sva package was used for removing batch effects and other unwanted variations. All the cases with survival status or follow‐up information missing were excluded.

### 3.2 Derivation of the gene expression signatures from TCGA data sets

**FIGURE 1:**
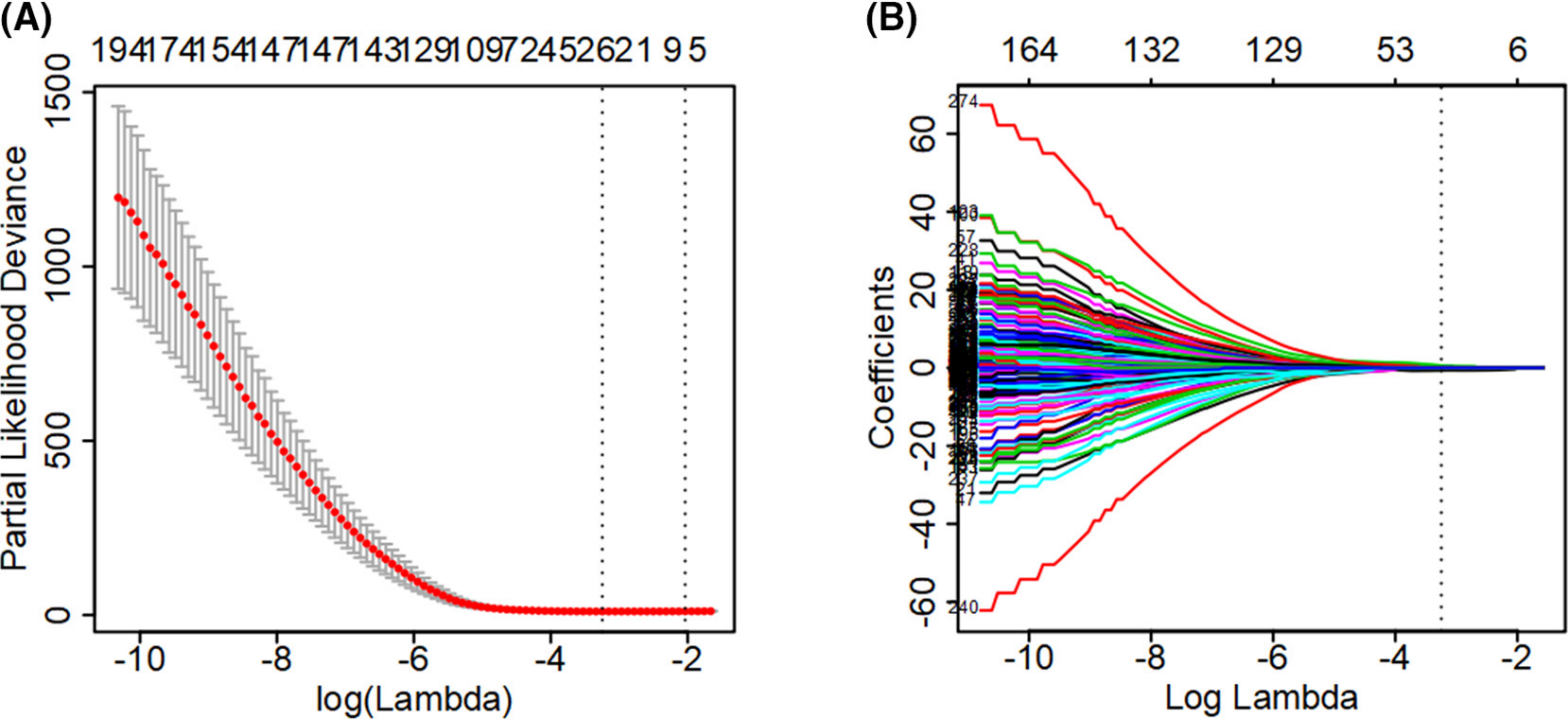
Analysis of autophagy‐related gene signatures in the TCGA cohort. (A) Tuning parameter (lambda) selection by the partial likelihood deviance, the lower partial likelihood deviance the better number of features in the LASSO regression model. (B) The penalty coefficient of 276 ARGs was optimized through 10‐fold cross‐validation in the training set

Altogether RNA‐seq and clinical data of 504 LGG samples were downloaded from TCGA. Clinicopathological features of these patients were described, especially IDH status and histopathological type (Table 1). After PS matching, there was no significant difference in age, gender, IDH status, histopathological type, and survival rate between training and test sets (Table 1). About 276 of 483 ARGs were found related to prognosis using Cox proportional hazards regression modeling, *p* < 0.05 was perceived as statistically significant (Supplementary Material S1 and S2). Furthermore, 24 out of 276 ARGs were found related to prognosis by performing LASSO method for a quantile regression model. This method possesses the oracle property and outperforms available existing approaches in many of the operating characteristics (Figure 1A,B). Maximum likelihood estimation picked 24 ARGs as significant covariates, and the coefficient estimated from the proposed shrinkage method‐ LASSO (Table 2). We derived a 24 ARGs signatures to calculate the predict index for each patient based on the expression levels of the 24 genes weighted by their regression coefficients: predict index = (−0.16347996*expression level of BAG1) + (0.31938936*expression level of LRRK2) + (0.09780923*expression level of ITGA3) + (0.05410868*expression level of DIRAS3) + (0.74255987*expression level of FGF7) + (−0.18145619*expression level of KCNK3) + (−0.03412787*expression level of BNIP3) + (0.06458334*expression level of CAPN1) + (0.06828578*expression level of DLC1) + (0.13859764*expression level of NFE2L2) + (−0.31072756*expression level of PTK6) + (−0.01122885*expression level of ACBD5) + (0.03437062*expression level of HIST1H3D) + (−0.89303388*expression level of SAR1A) + (0.30253164*expression level of SMURF1) + (0.00364621*expression level of HIST1H3H) + (−0.18239344*expression level of RNF185) + (0.02510785*expression level of ANXA5) + (−0.03842213*expression level of PEA15) + (−0.10924460*expression level of RRAGA) + (−0.10734054*expression level of NRG3) + (0.34811122*expression level of ERBB2) + (0.07805519*expression level of TP73) + (0.03900696*expression level of RBM18).

**TABLE 2:** Coefficients of the 24 ARGs signatures

Gene symbol	Coefficients
BAG1	−0.163479961
LRRK2	0.319389362
ITGA3	0.097809235
DIRAS3	0.054108684
FGF7	0.742559877
KCNK3	−0.181456197
BNIP3	−0.034127878
CAPN1	0.06458334
DLC1	0.068285784
NFE2L2	0.138597645
PTK6	−0.310727562
ACBD5	−0.011228855
HIST1H3D	0.034370624
SAR1A	−0.893033889
SMURF1	0.302531649
HIST1H3H	0.003646218
RNF185	−0.182393445
ANXA5	0.025107853
PEA15	−0.038422135
RRAGA	−0.109244609
NRG3	−0.107340544
ERBB2	0.348111222
TP73	0.078055191
RBM18	0.039006969

### 3.3 Validation of prognostic signatures

**FIGURE 2:**
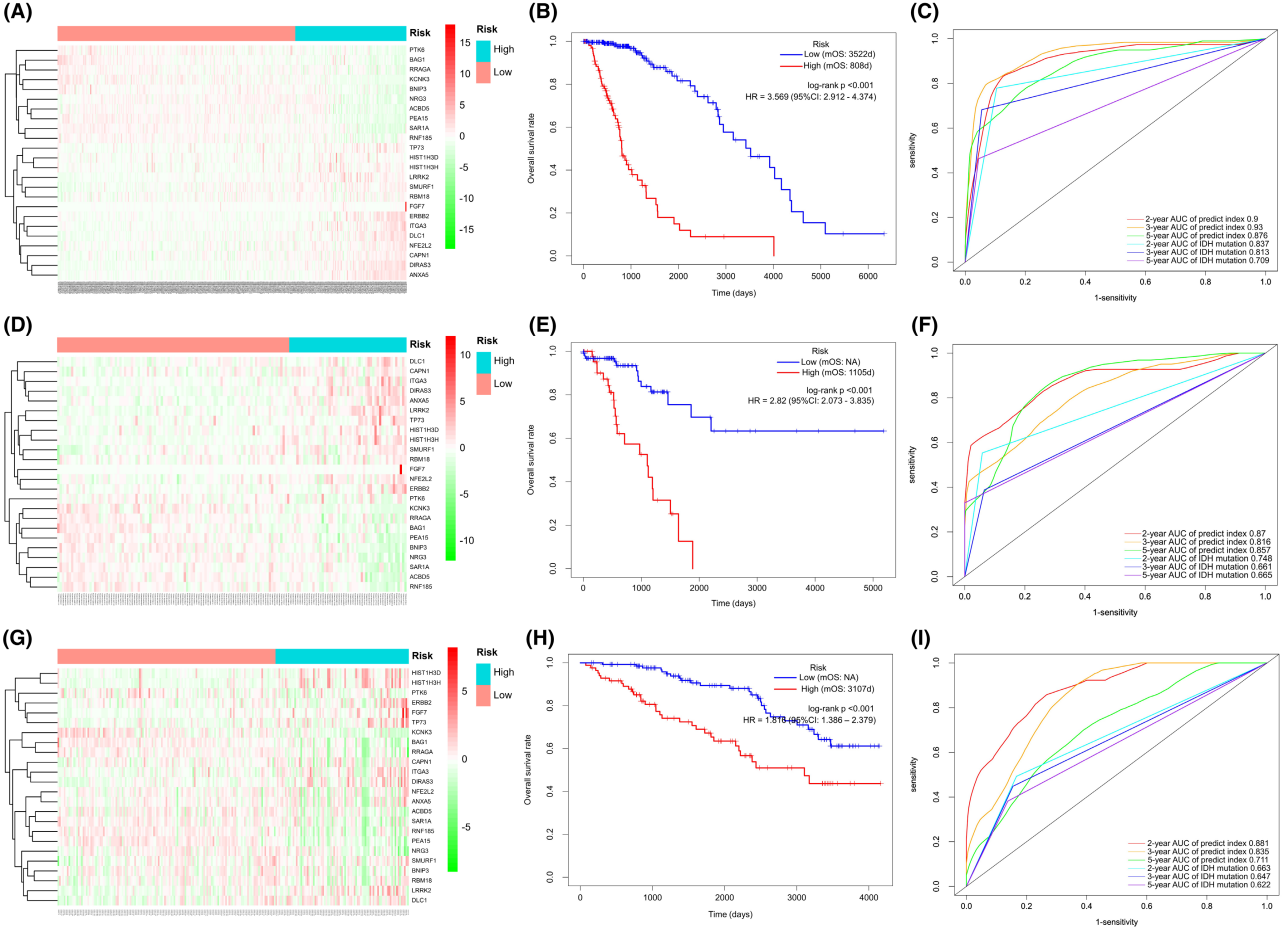
FIGUREDetermination and verification of 24‐autophagy‐related signatures in the TCGA and CGGA independent cohorts. The heatmap showed 24 differentially expressed autophagy genes in LGG between low and high groups of predict index in training set (A), test set (D) and validation set (G). Kaplan–Meier survival curves of overall survival rate between two clusters via Log‐rank in training set (B), test set (E), and validation set (H). Time dependent ROC curves between predict index and IDH mutation at 2, 3, and 5 years in training set (C), test set (F), and validation set (I). mOS: median overall survival

Patients in the TCGA training set use the median predict index as a cut‐off point to divide them into the low‐risk or high‐risk groups. The 24 ARGs expression is correlated with risk status in the heatmap (Figure 2A). The distribution of risk status and survival status were shown in Figure 2B, which suggested that low‐risk patients generally have better survival rates than high‐risk patients based on log‐rank test. Time‐dependent ROC analysis was conducted at 2, 3, and 5 years to assess the prognostic accuracy of the classifier based on 24‐ARGs. In TCGA training set, the AUC was 0.9, 0.93, and 0.876 at the survival time of 2, 3, and 5 years, respectively, which was better than the AUC of IDH mutation status (Figures 2C). The 24‐gene signature was tested for its prognostic significance in TCGA test cohort for internal verification. Using the established predict index formula, each case was divided into high‐risk or low‐risk group. We compared the ARGs expression between high‐risk and low‐risk groups in training and test sets. In both sets, the expression levels of LRRK2, ITGA3, DIRAS3, FGF7, CAPN1, DLC1, NFE2L2, HIST1H3D, SMURF1, HIST1H3H, ANXA5, ERBB2, TP73, and PRBM18 in the high‐risk group were higher than those in the low‐risk group, while BAG1, KCNK3, BNIP3, PTK6, ACBD5, SAR1A, RNF185, PEA15, RRAGA, and NRG3 were opposite (Figure 2D). Consistent with the above findings, log‐rank test analysis found that there were significantly different outcomes between the high‐risk group and the low‐risk group. The HR of overall survival rate of the high‐risk group and the low‐risk group were 3.569 (95% CI: 2.912–4.374, *p* < 0.001) and 2.82 (95% CI: 2.073–3.835, *p* < 0.001) in the training (Figure 2E) and test sets (Figure 2F), respectively. In the test set time‐dependent ROC analysis showed that at 2‐, 3‐, and 5‐year survival time, the AUC was 0.87, 0.816, and 0.857, respectively, better than the IDH mutation status AUC (Figures 2F).

### 3.4 External validation of the signatures in CGGA data sets

In addition to the original test cohort (TCGA), the external cohort CGGA met the inclusion criteria. The expression data of the 24 ARGs from these RNA‐seq data sets were extracted and the prognostic signatures based on the aforementioned formula were calculated. The heatmap that showing the relationship between 24 ARGs and risk groups was also displayed, which supported the findings of the testing cohort (Figure 2G). Survival differences between the high‐risk group and low‐risk group were assessed via the log‐rank test. In consistent with the above findings, patients from low‐risk and high‐risk groups showed significantly different outcomes (HR: 95% CI: –, *p* < 0.001) (Figure 2H). ROC analysis was used to investigate the prognostic or predictive accuracy of the signatures, which suggested that AUC was 0.881, 0.835, and 0.711, respectively, at 2‐, 3‐, and 5‐year survival time, while the IDH mutation status AUC was less than 0.7 at 2, 3, and 5 years. (Figure 2I).

### 3.5 Predict index was an independent prognostic factor

**FIGURE 3:**
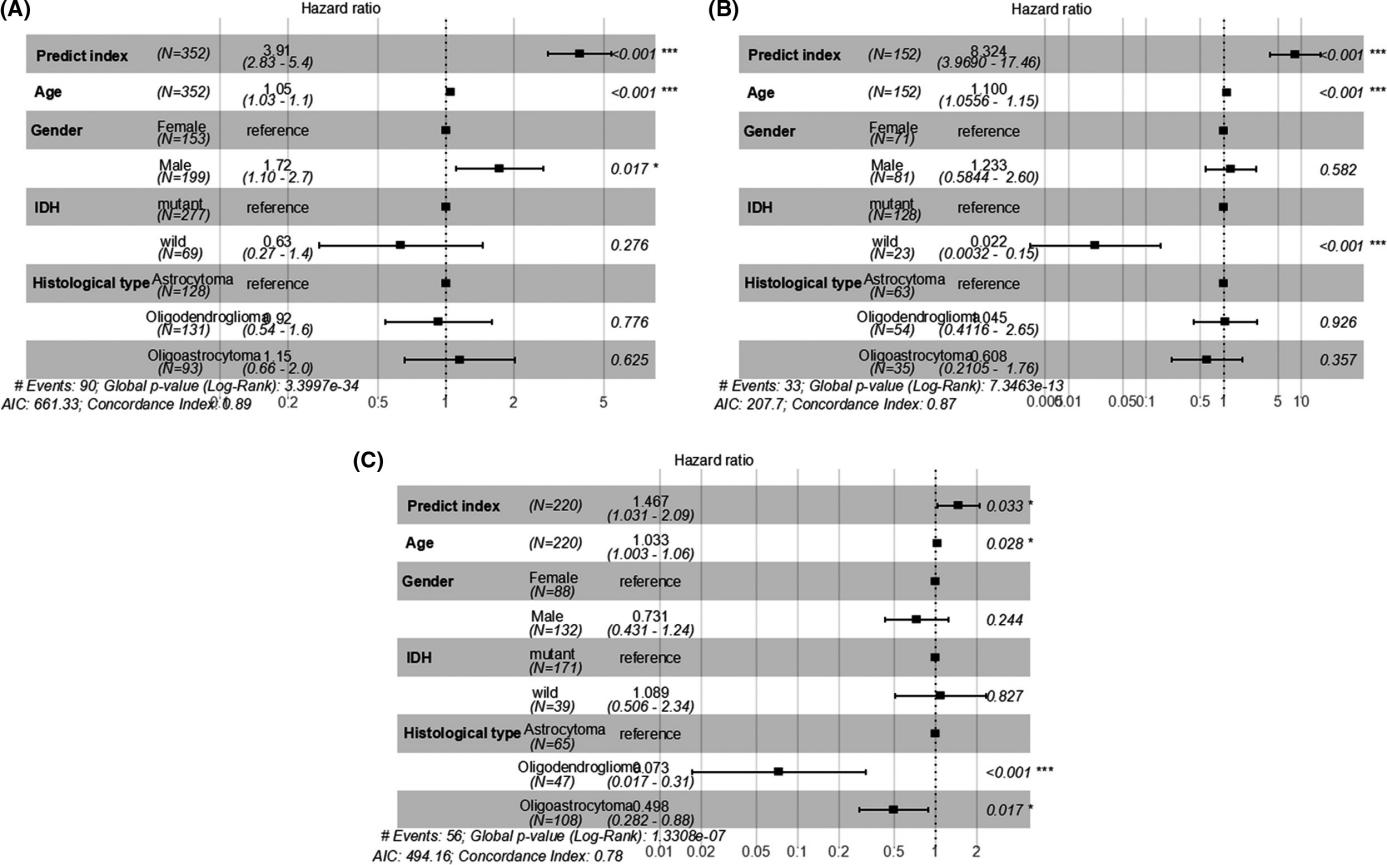
Multivariate Cox regression analysis of predict index and clinical parameters in TCGA and CGGA sets. Forest plot of HR in training set (A), test set (B), and validation set (C). HR: hazard ratio

In order to improve the robustness, predict index from 24 prognosis‐related ARGs were analyzed with clinicopathological features by univariate Cox (Table 3) and multivariate Cox regression model using SPSS 24.0. Furthermore, predict index and IDH1 mutation showed significant prognostic value with *p* < 0.001 in training, test and validation sets. However, predict index and age remained as independent prognostic indicator for the three sets in multivariate analysis, while IDH1 mutation and histological type were inconsistent in the three sets (Figure 3A‐C). Besides the parameters that we investigated, 1p19q codeletion and radiation were inconsistent in the three sets (Supplementary Material S3).

**TABLE 3:** Univariate Cox regression analysis of predict index and clinical parameters in TCGA and CGGA

Variables	TCGA cohort						CGGA cohort		
	Training set			Test set					
	Hazard ratio	*Z*‐score	*p*‐value	Hazard ratio	*Z*‐score	*p*‐value	Hazard ratio	*Z*‐score	*p*‐value
Predict index	3.568	12.265	<0.001	2.819	6.604	<0.001	1.815	4.329	<0.001
Age	1.056	6.343	<0.001	1.073	4.898	<0.001	1.029	1.949	0.051
Gender									
Female	—	—	—	—	—	—	—	—	—
Male	1.254	1.051	0.293	0.779	−0.721	0.470	0.748	−1.101	0.270
IDH									
Mutation	—	—	—	—	—	—	—	—	—
Wild	6.849	8.676	<0.001	5.009	4.111	<0.001	2.147	2.396	0.016
Histological type									
Astrocytoma	—	—	—	—	—	—	—	—	—
Oligodendroglioma	0.499	−2.84	0.004	0.844	−0.453	0.650	0.064	−3.741	<0.001
Oligoastrocytoma	0.711	−1.24	0.211	0.534	−1.216	0.223	0.454	−2.939	0.003

### 3.6 Construction of a nomogram for predicting prognosis model

**FIGURE 4:**
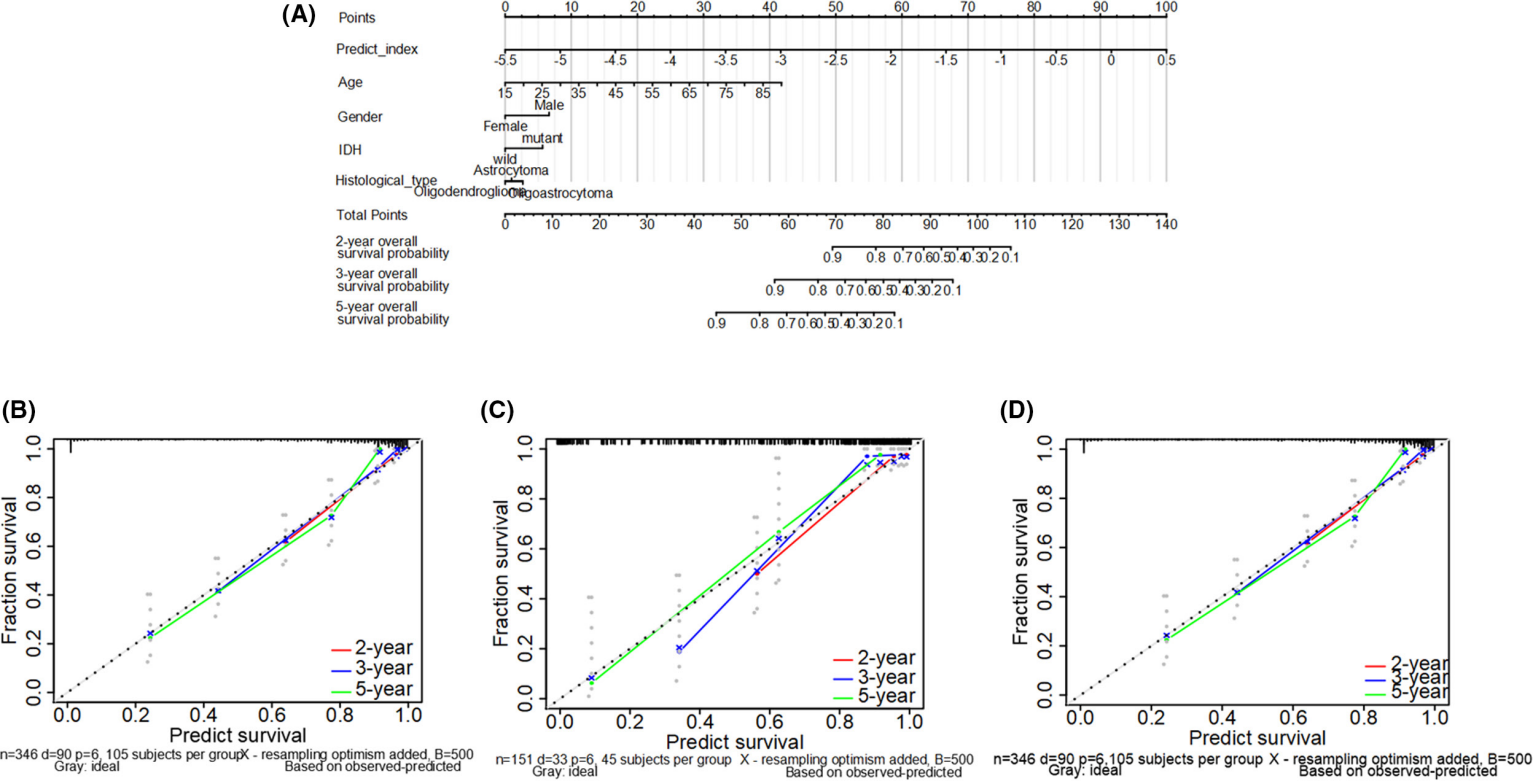
The construction and verification of Nomogram for predicting OS of patients of LGG patients using the predict index and four clinicopathological characteristics to convey the results of prognostic models. (A) Each parameter got the point at the top scale, and the total points can be converted to predict 2‐, 3‐, and 5‐year probability of OS in the lowest scale. The *x*‐axis is nomogram‐predicted survival and *y*‐axis is fraction survival. The reference line is 45◦ and indicates perfect calibration, in training set (B), test set (C), and validation set (D)

To provide a quantitative method for predicting prognosis, integrating the predicted index and four clinicopathological risk factors, a nomogram was constructed to meet the needs of clinicians. The nomogram can be interpreted by summarizing the points of the five variables, which were indicated at the top of the scale. The total points can be converted to patients with 2‐, 3‐, and 5‐year survival rates (Figure 4A). The predictive accuracy of the nomogram is investigated via the time‐dependent ROC, which suggested that the nomogram had amazing prognostic accuracy. Calibration curves for the nomogram revealed no deviations from the reference line and recalibration was out of consideration, whether it is in the training set, test set, or validation set. (Figure 4B–D).

### 3.7 Identification of the biological pathways associated to 24 ARGs

**FIGURE 5:**
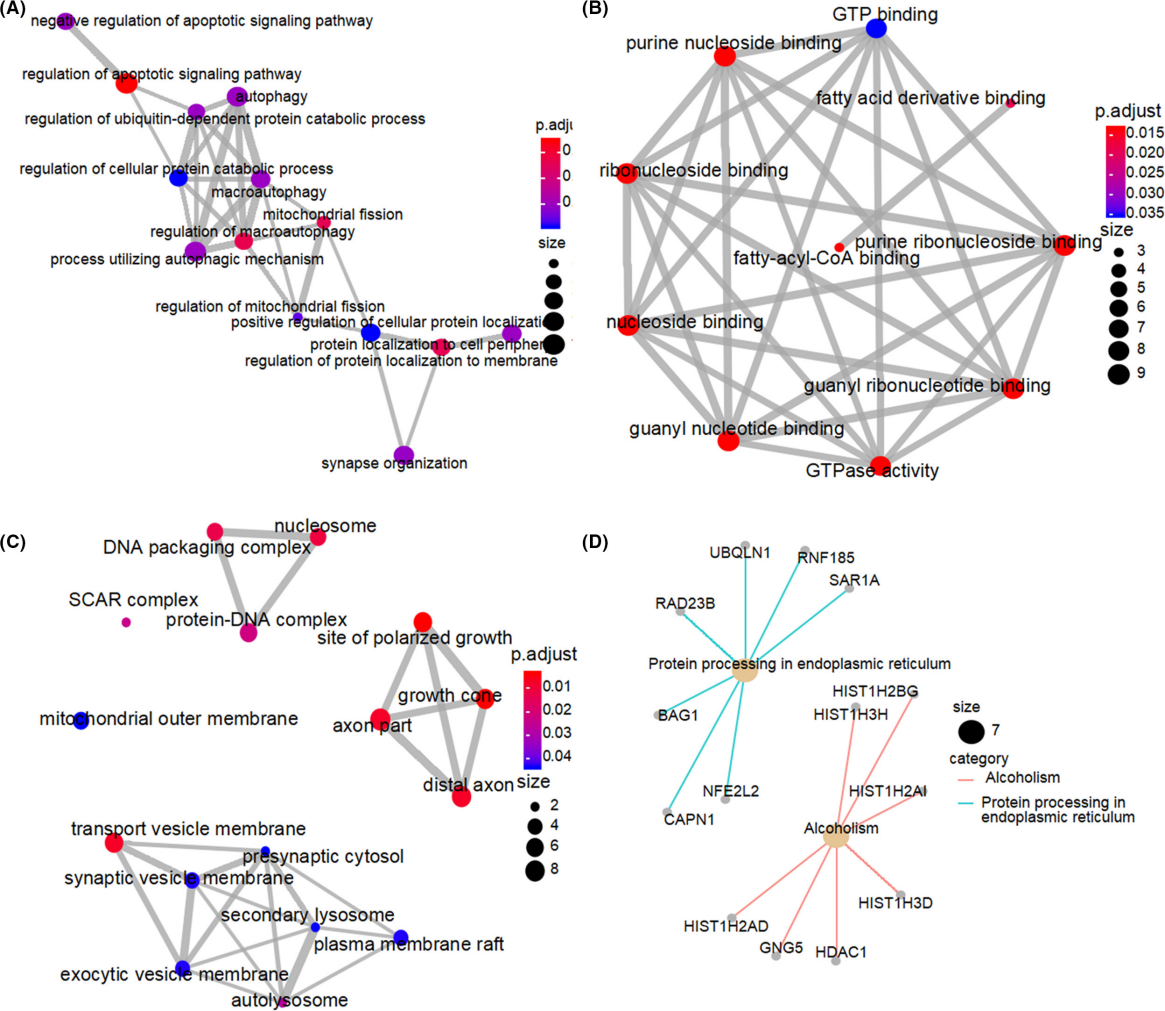
Network of the 24‐ARGs associated biological pathways. The size and redness of the circle represents the degree of connection. (A) Biological process. (B) Molecular function. (C) Cellular components. (D) KEGG

The GO terms function and KEGG pathway enrichment of these genes were analyzed to the biological understanding of the 24 ARGs (*p* < 0.05, |*r*| > 0.7). For GO terms of the biological process (BP), the top three terms were “regulation of apoptotic signaling pathway,” “regulation of macroautophagy,” and “protein localization to cell periphery” (Figure 5A). For GO terms of the molecular function (MF), the most important terms were “purine nucleoside binding,” “ribonucleoside binding,” “nucleoside binding,” “uanyl nucleotide binding,” “GTPase activity,” “guanyl ribonucleotide binding,” and “purine ribonucleoside binding” (Figure 5B). And for GO terms of the cellular component (CC), the tops terms were “nucleosome,” “site of polarized growth,” “axon part,” “growth cone,” “distal axon,” and “transport vesicle membrane” (Figure 5C). Accordingly, genes related to KEGG pathways included “Protein processing in endoplasmic reticulum” and “Alcoholism” activity (Figure 5D).

### 3.8 Correlation between predict index and clinic‐pathological features

**FIGURE 6:**
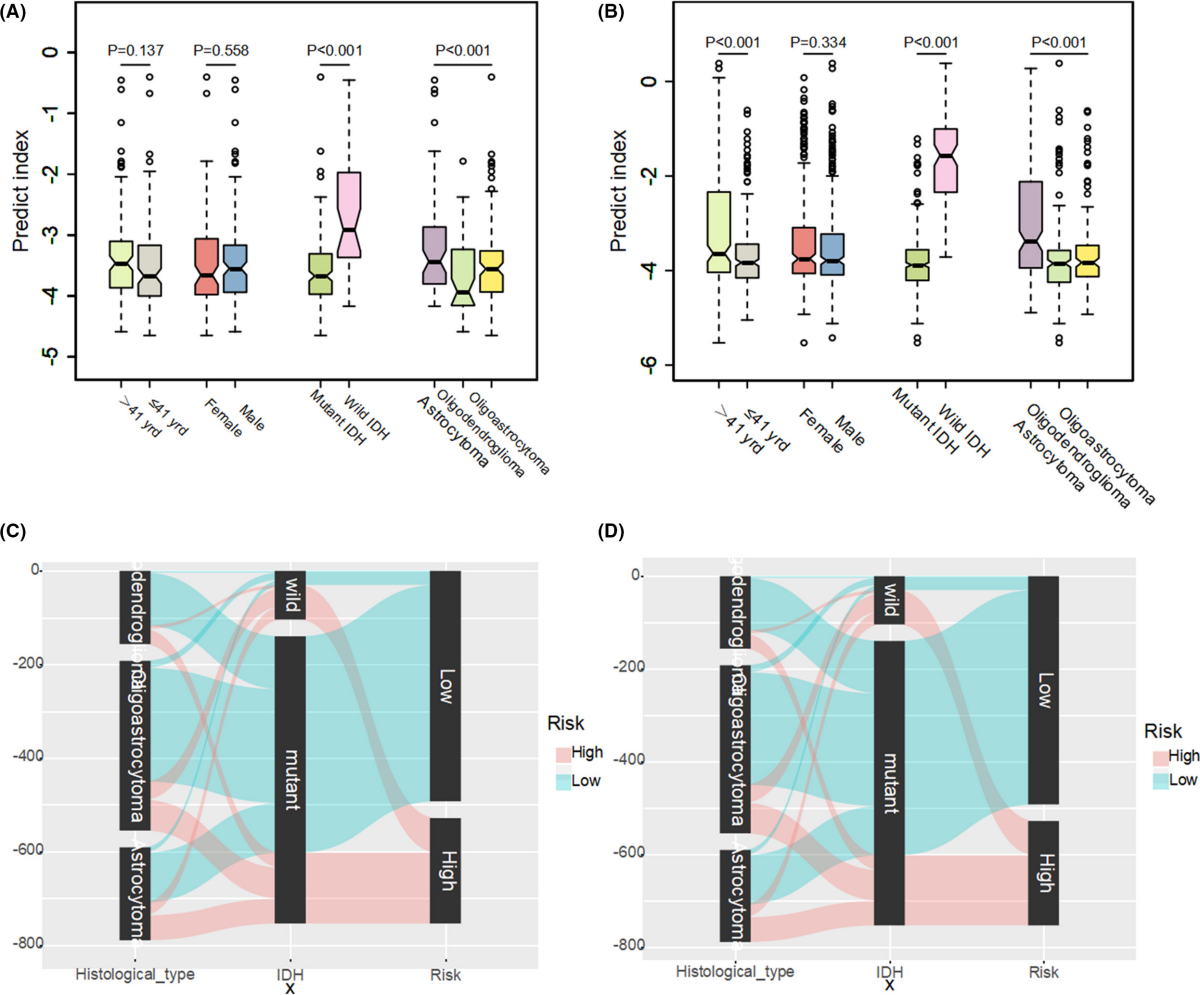
The correlation between predict index and clinicopathological parameters in LGG. Predict index across different clinicopathological parameters via independent samples nonparametric tests in TCGA cohort (A) and CGGA cohort (B). Data are presented as box plots where the box indicates percentiles 25th and 75th. Box line represents sample median and diamonds sample mean, notches mark the half‐width. Sankey diagrams in TCGA cohort (C) and CGGA cohort (D). Left column of Sankey diagrams: histological type (red: predict index high, green: predict index low). Middle column: IDH status. Right column of Sankey diagrams: predict index

**FIGURE 7:**
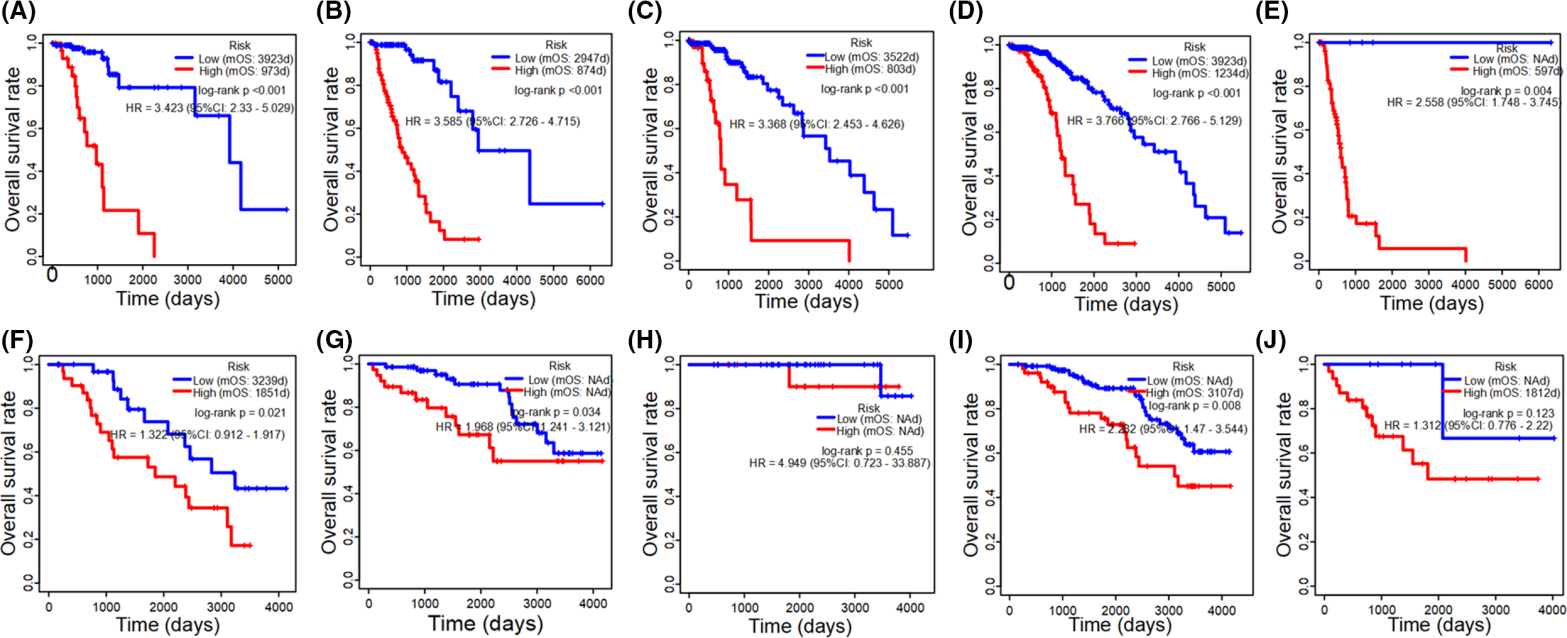
Kaplan–Meier survival curves of overall survival rate between two clusters (high‐risk and low‐risk) in different clinicopathological parameters. Kaplan–Meier plots summarize results from analysis of overall survival rate between high risk and low risk in oligoastrocytoma in the TCGA cohort (A), high‐risk and low‐risk in astrocytoma in TCGA cohort (B), high‐risk and low‐risk in oligodendroglioma in TCGA cohort (C), high‐risk and low‐risk in IDH mutation in TCGA cohort (D), high‐risk and low‐risk in IDH wild in TCGA cohort (E), high‐risk and low‐risk in oligoastrocytoma in CCGA cohort (F), high‐risk and low‐risk in astrocytoma in CCGA cohort (G), high‐risk and low‐risk in oligodendroglioma in CCGA cohort (H), high‐risk and low‐risk in IDH mutation in CCGA cohort (I), high‐risk and low‐risk in IDH wild in CCGA cohort (J). NA: not available. mOS: median overall survival

To investigate the correlations between predict index and clinicopathological parameters in LGG, multiple independent samples of nonparametric tests were used. IDH mutation and histological types were correlated with predict index, significantly, both in TCGA and CGGA cohorts (*p* < 0.001). Wild IDH and histological type with astrocytoma linked with higher predict index (Figure 6A,B). To visualize clinicopathological parameters and predict index calculated from 24 ARGs expression as one unified system, Sankey diagrams constructed to descript the relationship of histological type, IDH mutation and predict index. Most oligodendroglioma and oligoastrocytoma carried IDH mutation with low‐predict index, while most IDH wild type correspondence with high‐predict index both in the TCGA and CGGA cohorts (Figure 6C,D). However, patients with the same histological type or IDH status may have different predict index, which indicated that histological type and IDH status cannot predict prognostic effectively. Furthermore, differences between the high‐risk and low‐risk groups in different histological typed or IDH status were assessed via the log‐rank test. In TCGA cohort patients with oligoastrocytoma, astrocytoma, oligodendroglioma as well as IDH wild or mutation, showed significantly different outcomes between low‐risk and high‐risk groups (Figure 7A–E), while in CGGA cohort patients just with oligoastrocytoma, astrocytoma, and IDH mutation showed significantly different outcomes. In CGGA cohort, patients with oligodendroglioma and IDH wild did not show statistically difference in overall survival rate between low‐risk and high‐risk groups, which were not like TCGA cohort. To analyze potential mechanism, less than 50% death and small patients involved in oligodendroglioma and IDH wild cohorts were to blame(Figure 7F–J).

## 4 DISCUSSION

The role of autophagy in tumors has gradually been revealed, however, the expression of autophagy‐related genes in LGG is still a continuing controversy.^12^, ^19^, ^25^, ^26^ In the present study, ARGs expressions were analyzed from the TCGA and CGGA cohorts, and then, the prognostic value was subsequently examined. Finally, a predict index formula was constituted by using the 24 prognostic ARGs. Furthermore, the nomogram based on predict index and clinic‐pathological characteristics could well predict the clinical prognosis of patients in LGG. Given the clinical significance of these prognostic ARGs in LGG, if more aggressive treatment or drugs targeting gene expression were carried out, it may provide novel directions for LGG treatment. With the assistance of the TCGA training cohort, the expression of all 483 ARGs in LGG was evaluated. Univariate Cox analysis identified 276 prognostic ARGs, which indicated that autophagy plays an essential role in the process of LGG and influence the outcome of LGG patients. When performing the LASSO regression model, 24 out of 276 ARGs which were believed most relevant to prognostic were identified. Then, we built a predict index formula based on 24 autophagy‐related genes. When applying the 24‐ARGs signatures to LGG patients, a significance separation was observed in the Kaplan–Meier survival curves between low‐risk and high‐risk patients. Besides, it has been successfully verified in the TCGA test set and the external cohort of CGGA database, indicating good reproducibility. Furthermore, the time‐dependent ROC suggested that the 24‐ARGs signatures have a relatively high‐prognostic accuracy in predicting tumor relapse in the second, third, and fifth years of LGG patients’ survival. Further investigation found that predict index was an independent prognostic factor via multivariate Cox regression analysis. Meeting the needs of clinicians, a nomogram was conducted based on various parameters that we believed important in LGG's overall survival. To confirm whether the nomogram can be expanded to more patients, validation analysis was performed in the TCGA test set and the external cohort of CGGA database. In addition to autophagy, multiple extra functions of ARG have also been discovered, analyzed by GO and KEGG pathway, which including “purine nucleoside binding,” “ribonucleoside binding,” “nucleosome,” and “site of polarized growth.” For KEGG pathways, the “protein processing in endoplasmic reticulum” and “alcoholism activity” was identified. It has been documented that there was a close connection between protein processing in endoplasmic reticulum signaling and autophagy, and between alcoholism signaling and autophagy. When coming to the endoplasmic reticulum stress (ERS), the damaged endoplasmic reticulum can be engulfed by the autophagic vesicles.^27^ Ethanol‐induced neuron apoptosis by activating autophagy in the developing brain.^28^ In general, the signaling pathway of autophagy activation is still unclear. In this work, we conduct a set of visualization design requirements relating to Sankey diagrams. With a visualization approach, the relationship of the histological type, IDH status, and predict index were shown clearly. From the Sankey diagrams, patients with the same histological type or IDH status may have different predict indexes. High‐predict index indicates high‐risk shown shorter survival rate in the same histological typed or IDH status except in oligodendroglioma and IDH wild of CGGA cohorts. For most LGG accompanied with IDH mutation, sample size of IDH wild patients were small in the CGGA cohort, overall survival rate did not show statistically difference between low‐risk and high‐risk groups which was not like TCGA cohort. Besides, since low‐risk groups linked with longer survival rates, patients in the low‐risk group always prone to have less than 50% death, that is the main reason why there were no statistically differences between low‐risk and high‐risk groups in CGGA cohort with oligodendroglioma or IDH wild.

Since prognostic evaluations are performed based on 24 ARGs, our knowledge of the general overall survival has greatly improved, leading to a better‐personalized estimation of prognosis. It should be noted that this study was examined based on public data lacking several important clinicopathological features, such as specific tumor location, tumor size, extent of resection, KPS score, seizures, etc., and has not been prospectively tested in clinical trials.^3^ Besides, the underlying mechanism of how the identified 24‐ARGs play a role in the poor prognosis of LGG still requires further research.

## 5 CONCLUSION

In conclusion, we have developed a 24‐ARGs signatures that can effectively divide LGG patients into low‐risk and high‐risk groups for prognostic prediction. The signatures should be further applied in the clinic to verify our findings.

## CONFLICT OF INTEREST

The authors declare that the research was conducted without any potential conflict of interest.

## AUTHOR CONTRIBUTIONS

Jian Chen and Xiaojun Qian had the idea for this study. Yuntian Li supervised the acquisition of the data. Xinghua Han and Yueyin Pan undertook the statistical analysis. All authors contributed to interpretation of the results. Jian Chen and Xiaojun Qian wrote the article and other authors contributed to the content. All authors approved the final version of the manuscript.

## Supporting information

Supplementary Material

Supplementary Material

Supplementary Material

## Data Availability

The data of LGG in this study were downloaded from TCGA and CGGA databases. All autophagy‐related genes were obtained from the Human Autophagy Database.
