# Supplementary material for: An autophagic gene‐based signature to predict the survival of patients with low‐grade gliomas
Source: Cancer Med. 2021 Feb 16;10(5):1848–59. doi: 10.1002/cam4.3748 (PMC7940225; doi:10.1002/cam4.3748)
Supplement: Supplementary file 3 — Supplementary Material [file CAM4-10-1848-s003.docx]

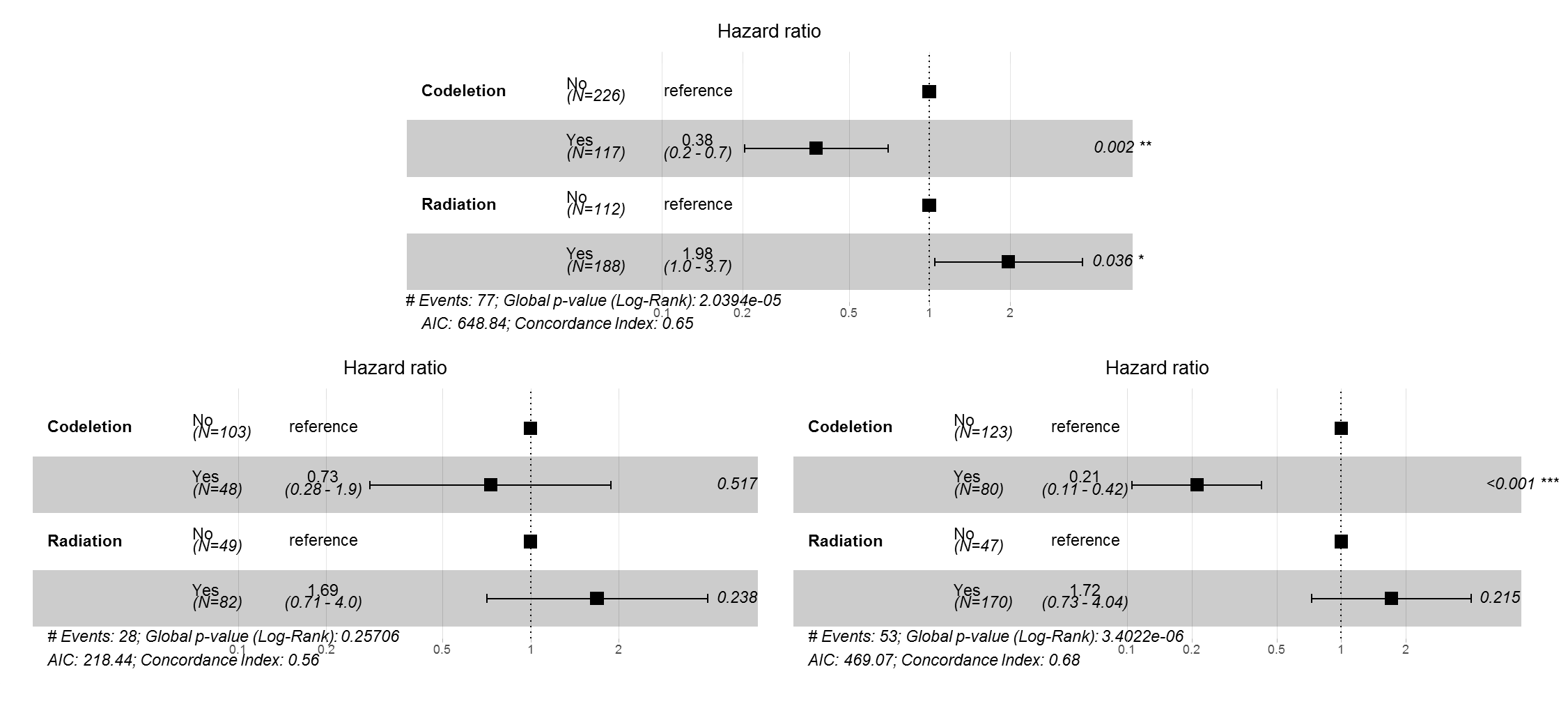


Supplementary 3 Multivariate Cox regression analysis of 1p19q codeletion and radiation in TCGA and CGGA sets. Forest plot of HR in training set (A), test set (B), and validation set (C). HR: hazard ratio.
